# Biocultural conservation trail system reduces bryophyte richness but not diversity in the southernmost miniature forest of the world.

**DOI:** 10.17912/micropub.biology.001307

**Published:** 2025-03-15

**Authors:** Morghan McCool, Clarissa Molina, Lucas Oyarzún Contreras, Sebastian Zambrano Rojas, Carmen Burkett, Francesca Burkett, Ri Corwin, Issabella Serrani Gellego, Benton J Hendrickson, Desiree Jackson, Sara Joseph, Jonathan Lautenbach, Erin Todd, Felipe Morales Armijo, Michael Thompson, Laura Sanchez Jardon, Roy Mackenzie, Andrew Gregory

**Affiliations:** 1 Dept. of Biology, University of Louisville, Louisville, Kentucky, United States; 2 Dept. of Biological Sciences, University of North Texas, Denton, Texas, United States; 3 Cape Horn International Center; 4 School of Earth Systems and Sustainability , Southern Illinois University Carbondale, Carbondale, Illinois, United States; 5 Dept. of Biological Sciences , Northern Arizona University, Flagstaff, Arizona, United States; 6 The Meadows Center , Texas State University, San Marcos, Texas, United States; 7 Ecosystem Science and Management, University of Wyoming, Laramie, Wyoming, United States; 8 School of Forestry, Northern Arizona University, Flagstaff, Arizona, United States; 9 Dept. of Philosophy and Religion , University of North Texas, Denton, Texas, United States; 10 Universidad de Magallanes, Punta Arenas, Region of Magallanes, Chile

## Abstract

The Cape Horn region of southern Chile is one of the remaining bryophyte (mosses, liverworts, and hornworts) hotspots in the world. The Omora Ethnobotanical Park on Navarino Island contains impressive examples of the region’s bryophyte diversity. A new trail has been proposed and we aimed to predict how a trail expansion might impact bryophyte communities. We compared the current trail and the proposed trail site and found significant differences. Specifically, there was no significant difference in bryophyte cover and diversity, but richness was lower at the existing trail. These findings indicate that ecotourism trails may negatively impact bryophyte communities.

**Figure 1. Differences between sampling sites at Omora Ethnobotanical Park:**
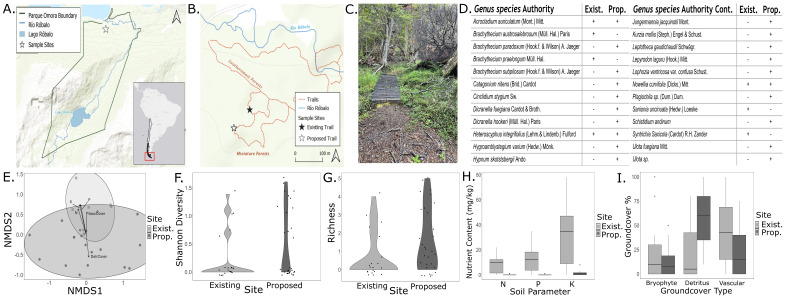
**A.** Map of Omora Ethnobotanical Park, Navarino Island where the location of the sample sites is denoted with a star. **B.** Trail map displaying the two bryophyte sampling sites at the existing and proposed trail at Omora Ethnobotanical Park. **C.** Photograph taken along the Miniature Forest Trail. **D.** List of bryophyte taxa and their presence or absence from each site. **E.** NMDS ordination plot displaying the concentrations of nitrogen, phosphorus, and potassium between the proposed and existing trail sites. **F.** The Shannon diversity index of both the existing and proposed trail site. **G.** Bryophyte species richness at the existing and proposed trail site. **H.** The average nutrient content of each nutrient measured (nitrogen, phosphorus, and potassium) at the existing and proposed trail site. **I.** The percent coverage of each groundcover type at the existing and proposed trail site.

## Description

Bryophytes (mosses, liverworts and hornworts) are the second most diverse group of land plants and exhibit high microhabitat specificity according to substrate quality, chemistry, moisture, and light (Mishler 2001). The Cape Horn Biosphere Reserve is one of the world’s few hotspots for bryophyte diversity, with around 5% of bryophyte diversity occurring in less than 0.01% of the planet’s surface (Goffinet et al. 2012). Additionally, bryophytes have high cultural significance to the Yaghan culture of indigenous people inhabiting the southern horn of South America (Rozzi and Schüttler 2013). As a result, bryophytes are protected by the Chilean government and are a priority for conservation and environmental attention throughout this region (Goffinet et al. 2012).

The Omora Foundation is dedicated to environmental stewardship and biocultural conservation of the Cape Horn region (Institute of Ecology and Biodiversity). As an extension of this commitment, the Omora Ethnobotanical Park was founded on Navarino Island to serve as the center for research, education, and conservation in the Cape Horn region (Goffinet et al. 2012). The park contains 1,000 hectares of protected austral forest, interpretive trails, and a portion of the Robalo river, an important source of drinking water for the residents of Puerto Williams, Navarino Island (Institute of Ecology and Biodiversity; Fig. 1A ).

In an effort to increase awareness of the region’s incredible bryophyte diversity, the Miniature Forest Trail was created. The name ‘Miniature Forest’ is a community outreach convention used to reference the complex microenvironment bryophytes provide to smaller organisms. The trail is used for ecotourism and environmental outreach as part of the Tracing Darwin’s Path program. As part of the education and outreach program, park visitors are encouraged to walk the trail system, get “nose-to-nose” with nature, and experience the diversity of non-vascular plants found throughout this region of the world. Both the park and program have been in operation since 2005, and hosts many school groups and hundreds of visitors annually (however, precise records are not maintained).

In November 2022, a large lenga tree ( *Nothofagus pumilio* ) collapsed and destroyed a portion of the Miniature Forest Trail. This fall created a large opening in the canopy and has caused the desiccation of bryophytes that were previously sheltered from water and light stress. In response, park managers have proposed a new trail to reconnect the severed trail halves. The goal of this study was to use the current Miniature Forest trail to make predictions of the putative impacts that a new or expanded trail system might have on bryophyte communities. This research also serves as a case study for assessing the consequences of ecotourism for regional bryophytes. Environmental impacts in the Cape Horn region are of local concern due to the recent expansion of ecotourism and the planned opening of Yendegaia National Park in 2030.

Here, we compare the cover of bryophytes and vascular plants, bryophyte species richness, and nutrient concentrations between existing trail sites and undisturbed forest sites proposed for the expanded trail system to assess the effect of trails on bryophytes. Trails are expected to increase available nitrogen, phosphorus, and potassium (NPK) concentrations, which will increase the cover of non-bryophyte vegetation. Soil compaction on dirt trails reduces permeability to water and dissolved nutrients which allows runoff to collect on either side of the trail (Adhikari and Bhattacharyya 2015). Bryophytes are not dependent on soil nutrients and will only be indirectly affected by increased NPK concentrations. Nutrient loading in areas otherwise known for being very nutrient-poor encourages the growth of vascular plants such as ferns and forbs that shade out most bryophytes (Adhikari and Bhattacharyya 2015). Forests lacking trails vary less in soil quality and are dominated by *Nothofagus* that provide enough shade to discourage the growth of vascular plants while supporting bryophytes (Stewart & Mallik 2006; Jägerbrand et al. 2012). In addition, management is expected to increase the diversity of vascular plants, but reduce the diversity of cryptogams (plants that reproduce via spores), including bryophytes (Kaufmann et al. 2017).

Over the course of two days, a total of 25 samples were collected. Eighteen were collected from the proposed site and seven from the existing site. Between both sites, 24 species were identified. Of the 20 species from the proposed site, 17 were unique ( Fig. 1D ). Of the seven species identified at the existing site, four were unique.

The NMDS indicated the community structure differed between sites, which may be associated with differences in ground cover and soil nutrition (stress = 0.0985, Fig. 1E ). The PERMANOVA indicated significant differences in community composition were associated with differences between sites (F = 2.036, p < 0.05), the interactive effect of site and phosphorus (F = 2.383, p <0.05), and the interactive effect of site, nitrogen, and phosphorus (F = 2.2912, p <0.05).

There was no significant difference between Shannon diversity values for each site (U = 363.5, p > 0.05; Fig. 1F ). There was a significant difference between richness values for each site, with the proposed site hosting a greater richness than the existing site (U = 398.5, p<0.05; Fig. 1G ). Significant differences were found in nutrient concentrations between sites (N: U = 72, p <0.001; P: U = 74, p <0.001; K: U = 77, p <0.001) and the average NPK concentration was higher at the existing trail site than at the proposed site ( Fig. 1H ). There was no significant difference in bryophyte cover between sites (U = 271.5, p > 0.05; Fig. 1I ).

These results indicate a significant effect of trail use on bryophyte richness. Richness was significantly higher at the proposed trail site, but these differences were not correlated with NPK variation, and therefore likely not associated with changes in forest cover. The differences in phosphorus concentrations is interesting but not surprising as phosphorus is often the limiting factor for primary productivity because it is not readily available in the ecosystem, and assimilation is very slow (Kerkhoff et al. 2006). In addition, utilization of phosphorus by primary producers is directly dependent on nitrogen concentration (Elser et al. 2007; Ågren et al. 2012). This relationship is also evident in bryophytes (Dirske and Martakis, 1992), however, there is evidence that increases in NPK concentrations decrease bryophyte cover and richness, largely due to overcrowding by vascular plants (Virtanen et al. 2022). The lack of an effect of nitrogen on bryophyte cover was likely due to the role of bryophytes in the facilitation of fungi responsible for the decomposition of coarse woody debris (Glime 2017). However, the rate of decomposition by these fungi is limited by nitrogen. As a result, the availability of nitrogen to primary producers remains low until the needs of fungi have been satisfied (Oishi 2023).

The significant difference in species richness and lack of a significant difference in bryophyte diversity suggest that the existing trail site is lacking several species with few enough representatives that their absence doesn’t impact diversity estimates. The existence of trails therefore may have biologically significant implications for less common and rare bryophyte species. Rare species may be more ecologically sensitive to microhabitat changes expected to occur after trail development. To confirm and further elucidate the effects of human disturbance on these hyperdiverse ecosystems, long-term observation studies should measure the changes to those systems before and after frequent human exposure.

## Methods

*Field Methods*

To predict the diversity of bryophytes following ecotourism impacts, two sites were sampled - a “current existing trail” site and a “proposed trail” site 80 m to the southwest ( Fig. 1B ). The “proposed trail” is the proposed expansion of the Miniature Forest trail. Bryophytes were collected from a site along the existing trail relatively unaffected by the recent tree fall ( Fig. 1C ). At the existing trail, two 10-m transects were drawn over the ground extending from either side of the trail (10 m to the left, 10 m to the right, 20 m total). At even intervals along each transect (2-, 4-, 6-, 8-, and 10-m), left and right transects extended 1 meter from the trail. At odd intervals (1-, 3-, 5-, 7-, and 9-m), left and right transects extended 5 meters from the trail. At the proposed trail site, a large tree marked the vertex of the rounded trail. Starting at the tree, 10-meter transects were drawn to the north, south, and west. Congruent 5-meter transects were drawn to the left and right of the north and south transects at the 1-, 3-, 5-, 7- and 9-m distances. Congruent 1-meter transects were drawn to the left and right of the west transect at the 2-, 4-, 6-, 8-, and 10-m distances. Transects were not drawn to the east due to the proximity to the existing trail. Daubenmire frames were placed at the end of the transects to collect bryophyte samples, estimate groundcover, and measure nutrient concentrations (Daubenmire 1959). Nitrogen (N), phosphorus (P) and potassium (K) content (mg/kg) was measured using Fish Hawk Soil Parameter Probes. The probe was inserted into the ground at 3 random points within the frame and the average value was used to represent the nutrient concentration for each plot. Percent coverage of bryophytes, detritus, bare ground, and vascular vegetation were visually estimated within the frame. At each sample point, small portions of each unique bryophyte were sampled for identification in the lab. Bryophytes were identified to either genus or species using Buck & Goffinet (2010), Drapela & Larraín (2020), and Engel (1990). To identify a bryophyte to species, a thorough dissection of the sporophyte is often necessary for some groups. However, specimens lacking a sporophyte were identified only to the genus level to the inability to distinguish the gametophyte from other members of the genus. Thus, when assessing community structure, that generic identity (ex. Ulota sp.) included all of those plants indiscernible from other Ulota species. Canopy cover photos were taken at the 1-m and 10-m transect points at the existing site and the 10-m points for the north, south, and east transect points at the proposed site.

*Data Methods*

All statistical analyses were conducted in R (v4.3.3; R Core Team 2024). Multivariate analyses follow the methods outlined in Kutnar et al. (2023). Bryophyte communities were determined for each plot based on a presence-absence matrix of species. A matrix was constructed where each row corresponds to a plot, and columns contain the site information, ground cover percentages, NPK values, and a presence-absence matrix for species. The *diversity* function in the “vegan” package was used to calculate the Shannon Diversity Index for each plot, and richness was calculated using the sum of all species present in the plot.

The community composition matrix was analyzed using non-metric multidimensional scaling (NMDS), with environmental variables fitted to the ordination. The *metaMDS* function from the “vegan” package was used to generate the ordination space, and the *envfit* function was used to fit environmental variables to the community data. NMDS was performed using the Bray-Curtis dissimilarity index in a two-dimensional space (k = 2) to explore changes in community composition in response to environmental factors between the proposed and existing sites.

A Permutational Multivariate Analysis of Variance (PERMANOVA) is a multivariate technique that analyzes the partitioning of variance across different groups. The *adonis2* function in the “vegan” package was used to perform a PERMANOVA (with 9999 permutations) to assess differences in community structure after accounting for differences in site, NPK, and ground cover (CommunityMatrix ~ Site*N*P*K+DetritusCover+VascularCover). A Mann-Whitney U-test was used to further understand the differences in diversity, richness, ground cover, and soil nutrients between the two sites. A generalized linear model (GLM) was used to predict the effect of vascular plant ground cover and detritus ground cover on the bryophyte ground cover, diversity and richness.
